# Prognostic significance of TP53 and PIK3CA mutations analyzed by next-generation sequencing in breast cancer

**DOI:** 10.1097/MD.0000000000035267

**Published:** 2023-09-22

**Authors:** Jin Hyuk Choi, Jesang Yu, Minjung Jung, Junyong Jekal, Ku Sang Kim, Sung Ui Jung

**Affiliations:** a Division of Breast Surgery, Department of Surgery, Kosin University Gospel Hospital, Busan, Korea; b Kosin University College of Medicine, Busan, Korea; c Department of Radiation Oncology, Kosin University Gospel Hospital, Busan, Korea; d Department of Pathology, Kosin University Gospel Hospital, Busan, Korea.

**Keywords:** breast cancer, disease-free survival, next-generation sequencing, PIK3CA, somatic mutations, TP53

## Abstract

Breast cancer is one of the most prevalent malignant tumors affecting women globally. It is a heterogeneous disease characterized by mutations in several genes. Several gene panels have been applied to assess the risk of breast cancer and determine the appropriate treatment. As a powerful tool, Next-generation sequencing (NGS) has been widely utilized in cancer research due to its advantages, including high speed, high throughput, and high accuracy. In this study, we aim to analyze the correlation between somatic mutations in breast cancer, analyzed using NGS, and the prognosis of patients. Between May 2018 and May 2019, a total of 313 patients with breast cancer underwent surgical treatment, which included total mastectomy and breast-conserving surgery. Among these patients, 265 were diagnosed with invasive ductal carcinoma. In this study, we analyzed the NGS results, clinicopathological characteristics, and their correlation with prognosis. Using a gene panel, we examined 143 somatic mutations in solid cancers. Notably, the study population included patients who had received neoadjuvant chemotherapy. The mean age of the patients was 53.1 (±10.28) years, and the median follow-up time was 48 months (range, 8–54). Among the 265 patients, 68 had received prior systemic therapy. Of these, 203 underwent breast-conserving surgery, and 62 underwent a mastectomy. Various somatic mutations were observed in NGS, with the most frequent mutation being *PIK3CA* mutations, which accounted for 44% of all mutations. *TP53* mutations were the second most frequent, and *ERBB2* mutations were the third most frequent. *TP53* mutations were associated with poor disease-free survival (*P* = .027), while *PIK3CA* mutations were associated with better disease-free survival (*P* = .035) than *PIK3CA* wild-type. In our study, we identified various somatic mutations in breast cancer. Particularly, we found that TP53 and PIK3CA mutations are potentially associated with the prognosis of breast cancer. These findings suggest that the presence of specific mutations may have implications for predicting the prognosis of breast cancer. Further research and validation are needed to gain a deeper understanding of the role of these mutations and their mechanisms in prognosis prediction.

## 1. Introduction

Somatic mutations are mutations expressed in tumor cells. Many studies have been conducted to develop treatments that target somatic mutations, and their value as a prognostic factor is currently being investigated.^[1]^

Breast cancer is one of the most common malignant tumors affecting women worldwide. It is a heterogeneous disease characterized by mutations in several genes that affect its treatment and prognosis.^[2]^ The prognosis of breast cancer varies depending on whether the expression of hormone receptors and human epidermal growth factor receptor 2 (HER2) is positive or negative, which can be determined by immunohistochemical (IHC) staining.^[3]^ Each subtype of breast cancer distinguished by IHC has a different prognosis, and various mutations are expressed in tumor cells.^[4–6]^

Next-generation sequencing (NGS) and second-generation sequencing technology can run a large number of sequencing reactions in parallel, reducing the time and cost of genomic analysis. Several NGS panels have been developed and are used in a variety of clinical settings after genomic analysis.^[7]^ Efforts are currently underway to develop gene panels utilizing NGS for the purpose of predicting prognosis in breast cancer. Several gene panel studies have been developed and are being utilized to predict prognosis and guide the decision-making process for chemotherapy in estrogen receptor (ER)-positive, HER2-negative breast cancer.^[8]^ However, the precise significance of individual somatic mutations in this context has not yet been fully elucidated. Especially the mutation profile of operable breast cancer has not been clearly identified, and the clinical usefulness of NGS is still unclear.^[9]^

In this study, we performed NGS on operable breast cancer that occurred spontaneously for 1 year. We retrospectively analyzed the data through medical records, conducting a median 48-month (2–54) follow-up observation on patients who underwent NGS. We analyzed the genetic profile of breast cancers occurring in a region, identified the characteristics associated with clinicopathological factors, and studied the prognostic value of each mutation.

## 2. Method

### 2.1. Patient selection and data acquisition

**Figure 1.**
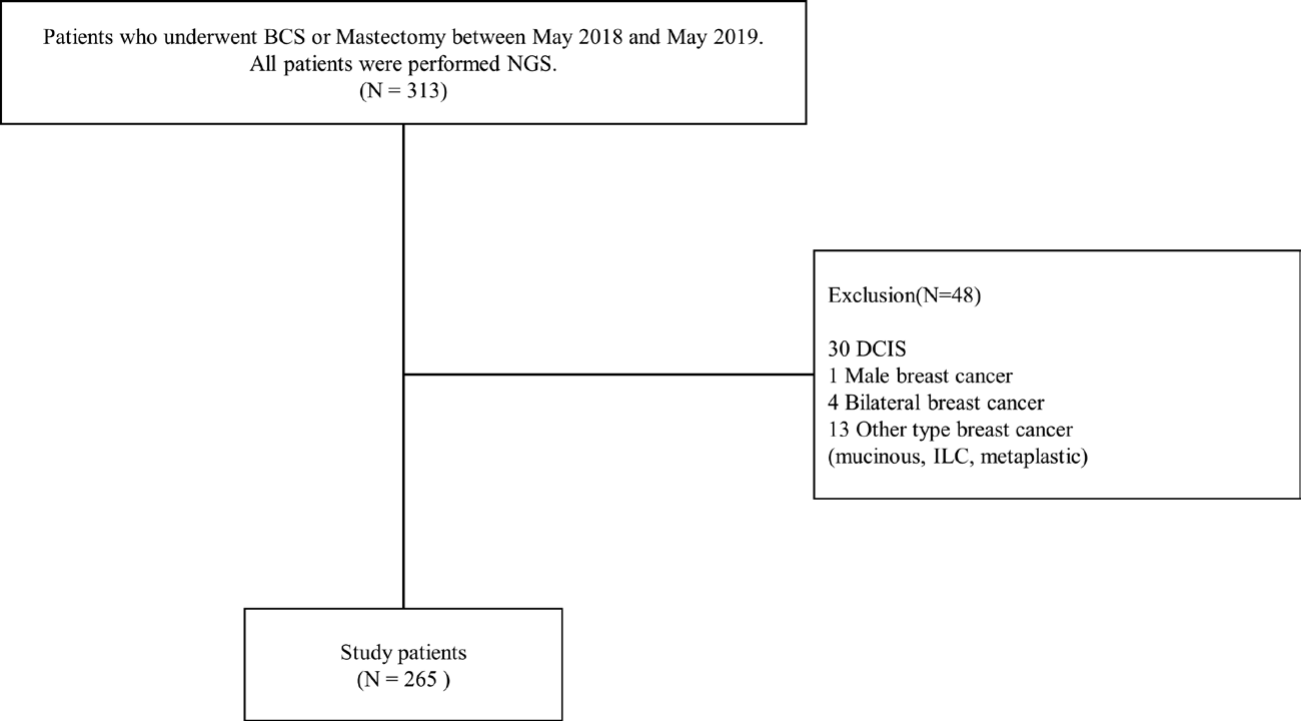
Diagram of inclusion and exclusion criteria. BCS = breast-conserving surgery, DCIS = ductal carcinoma in situ, ILC = invasive lobular carcinoma, NGS = next-generation sequencing.

Targeted NGS was initiated at Kosin University Gospel Hospital in May 2018. Between May 2018 and May 2019, 313 patients with breast cancer underwent surgical treatment, including total mastectomy and breast-conserving surgery at Kosin University Gospel Hospital. NGS was performed on all 313 patients with stages I to III breast cancer who were eligible for breast cancer surgery within 1 year. Among the 313 patients, 265 were diagnosed with invasive ductal carcinoma. Only invasive ductal carcinomas were analyzed; other types of breast cancer, such as mucinous, lobular, or papillary carcinomas, were excluded. Patients with carcinoma in situ, metastatic, and bilateral breast cancers were also excluded from the study (Fig. 1). A total of 48 patients were excluded from the study, and the analysis was conducted on 265 patients. Patients who underwent neoadjuvant chemotherapy underwent NGS of biopsies before receiving neoadjuvant chemotherapy. We retrospectively reviewed the electronic medical records of patients with breast cancer who underwent surgical resection between May 2018 and May 2019 at Kosin University Gospel Hospital. We collected clinicopathological data, including age at diagnosis, histological grade, tumor size, lymph node metastases, and IHC staining. Pathological data included the ER, progesterone receptor, HER2, and Ki-67 index. We classified the patients into 5 subtypes: luminal A, luminal B or HER2-negative, luminal B or HER2-positive, HER2-enriched, and triple negative breast cancers (TNBC) according to the Saint Gallen consensus.^[10]^ A cutoff value of 20% was adopted for Ki-67 to differentiate between luminal A and luminal B or HER2-negative subtypes.^[11]^ According to the NCCN guidelines, systemic therapy for breast cancer was administered based on the stage, ER/progesterone receptor status, and HER2 status. For all ER-positive breast cancer cases, endocrine therapy was performed. In ER-positive, HER2-negative cases, a gene panel study such as oncotype DX was conducted following the NCCN guidelines, and high-risk patients received chemotherapy. Node-positive or aggressive subtypes such as ER-negative or HER2-positive patients received chemotherapy, which consisted of taxane and anthracycline-based chemotherapy. In HER2-positive cases with a tumor size larger than 1 cm, adjuvant therapy with trastuzumab was utilized, and if node metastasis was detected, pertuzumab was added. Preoperative chemotherapy included taxane, carboplatin, trastuzumab, and pertuzumab for HER2-positive breast cancer patients. For all patients with TNBC, anticancer chemotherapy was performed.^[12]^ This retrospective study was conducted using de-identified data from patients who underwent surgical resection for breast cancer between May 2018 and May 2019 at Kosin University Gospel Hospitalon. The study protocol was reviewed and approved by the Clinical Review Board of the Kosin University Gospel Hospital (IRB number: 2022-11-003). As this study involved a retrospective design and utilized de-identified data, the requirement for informed consent was waived by the Institutional Review Board. Patient consent was not obtained directly for this study, and all patient data were handled confidentially following privacy regulations.

### 2.2. Tumor classification and staging

The tumor underwent histological classification following the guidelines provided by the World Health Organization. For tumor grading, the Nottingham modification of the Scarff-Bloom-Richardson grading system was utilized. The tumor staging was determined based on the anatomical staging system outlined in the 8th edition of AJCC, using the TNM system adopted by both the UICC and the American Joint Committee on Cancer and End Results Reporting. The oversight of tumor classification and staging was carried out by 3 experienced pathologists from the Department of Pathology at Kosin University Gospel Hospital.

### 2.3. NGS protocol

For NGS, we collected formalin-fixed, paraffin-embedded (FFPE) tumor-containing tissue blocks with at least 30% tumor content. DNA was isolated from cut sections of FFPE blocks using the All Prep DNA/RNA FFPE Kit (QIAGEN, Hilden, Germany). Cancer-related genes were selected (143 genes in 265 patients). Library preparation was performed using the Oncomine Comprehensive Assay V3 Panel (Thermo Fisher Scientific, Waltham, MA). We used Ion S5 systems (Thermo Fisher Scientific, Waltham, MA) for sequencing and Oncomine Comprehensive Plus – EA v3 RNA-w1.0 – DNA and fusions – Single Sample Workflow or Oncomine Comprehensive Plus – w2.3 – DNA – Single Sample Workflow for bioinformatics analysis, following the manufacturer’s instructions.

The genomic profiles generated were compared with known genomic variants provided in web-based databases, such as COSMIC (https://cancer.sanger.ac.uk/cosmic), GeneCards (https://www.genecards.org/), and ClinVar (https://www.ncbi.nlm.nih.gov/clinvar/).

The NGS panel included the following genes: *ALK, BRAF, EGFR, ERBB2, IDH1, IDH2, KIT, KRAS, NRAS, PDGFRA, MYC, MYCN, BRCA1, BRCA2, ABL1, ACVRL1, AKT1, AKT3, APC, APEX1, AR, ARAF, ATM, ATP11B, AXL, BAP1, BCL2L1, BCL9, BIRC2, BIRC3, BTK, CBL, CDK4, CHEK2, CSF1R, CTNNB1, CCND1, CCNE1, CD274, CD44, CDK6, CSNK2A1, CDH1, CDKN2A, DDR2, DNMT3A, DCUN1D1, ERBB3, ERBB4, ERG, ETV1, ETV4, ETV5, ESR1, EZH2, FGFR1, FGFR2, FGFR3, FGFR4, FLT3, FOXL2, FBXW7, GATA2, GATA3, GAS6, GNA11, GNAQ, GNAS, HNF1A, HRAS, IGF1R, IL6, IFITM1, IFITM3, JAK1, JAK2, JAK3, KDR, KNSTRN, MAGOH, MAP2K1, MAP2K2, MAPK1, MAX, MED12, MET, MLH1, MPL, MTOR, MYD88, MCL1, MDM2, MDM4, MYCL, MYO18A, MSH2, NFE2L2, NPM1, NKX2-1, NKX2-8, NF1, NF2, NOTCH1, NTRK1, NTRK3, PAX5, PIK3CA, PPP2R1A, PTPN11, PDCD1LG2, PNP, PPARG, PIK3R1, PTCH1, PTEN, RAC1, RAF1, RET, RHEB, RHOA, RPS6KB1, RB1, ROS1, SF3B1, SMO, SPOP, SRC, STAT3, SOX2, SMAD4, SMARCB1, STK11, TET2, TP53, TSC1, TSC2, TERT, TIAF1, U2AF1, VHL, WT1, XPO1,* and *ZNF217*.

### 2.4. Statistical analysis

Disease-free survival (DFS) was defined as the time from surgery to disease recurrence or death. The overall survival (OS) period was defined as the time from surgery to death from any cause.

The chi-squared (χ^2^) or Fisher exact test was used to compare the categorical variables. A Student *t* test was performed to compare continuous variables. The Kaplan–Meier curves were used to evaluate DFS and OS. A Cox proportional hazards model was used to calculate the adjusted hazard ratio for patient characteristics and other significant prognostic factors.

All reported *P* values were 2-sided, and a *P* value of < .05 was considered statistically significant. The results were analyzed using IBM SPSS Statistics for Windows, version 28.0 (IBM Corp., Armonk, NY).

## 3. Result

### 3.1. Patients and clinicopathological characteristics

A total of 313 patients underwent surgery for breast cancer between May 2018 and May 2019. Among the patients with breast cancer who underwent NGS, 265 were included in the analysis. The mean age was 53.1 (±10.28) years, and the median follow-up time was 48 months (range, 8–54). The stage, histological grade, and IHC results were collected from the study group. Of the 265 patients, 68 had received prior systemic therapy. Breast-conserving surgery was performed on 203 patients, while mastectomy was performed on 62 patients (Table 1).

**Table 1:** Characteristics of the study population and tumors.

Characteristics	Status	N = 265(%)
Age		53.1 ± 10.28
Median f/u time		48 (8–54)
Age	<50	113 (12.6)
	≥50	152 (57.4)
T stage	1	176 (66.4)
	2	87 (32.8)
	3	2 (0.8)
N stage	0	215 (81.1)
	1	41 (15.5)
	2	8 (3.0)
	3	1 (0.4)
Histologic grade	1	57 (21.5)
	2	123 (46.4)
	3	61 (23.0)
	Unknown	24 (9.1)
ER	Positive	182 (68.7)
	Negative	81 (30.6)
PR	Positive	163 (61.5)
	Negative	100 (37.7)
HER2	Positive	59 (22.3)
	Negative	204 (77.0)
Ki-67	<10%	102 (38.5)
	10–20%	68 (25.7)
	>30%	95 (35.8)
NST	Yes	68 (25.7)
	No	197 (74.3)
Subtype	Luminal A	119 (45.2)
	Luminal B (her2 negative)	39 (14.8)
	Luminal B (her2 positive)	25 (9.5)
	Her2 enriched	32 (12.2)
	TNBC	48 (18.3)
Op type	Mastectomy	62 (23.4)
	BCS	203 (76.6)

Values are expressed as mean ± standard deviation or number (%).

BCS = breast-conserving surgery, ER = estrogen receptor, HER2 = human epithelial growth factor receptor 2, NST = Neo-systemic therapy, PR = progesterone receptor, TNBC = triple negative breast cancer.

*TP53* mutations were significantly associated with poor clinicopathological characteristics, including hormone receptor negativity, HER2 positivity, and high histological grades (Table 2). Contrastingly, *PIK3CA* mutations were associated with better histological features, such as a lower histological grade, hormone receptor positivity, and lower Ki-67 levels (Table 3).

**Table 2:** Comparison of clinical and molecular characteristics between TP53 wild-type and mutant breast cancer.

Variables		*TP53* wild-type (N = 160)	*TP53* mutant (N = 105)	*P* value
Age		53.28 ± 10.69	52.94 ± 9.66	.082
median f/u time		48 (8–54)	49 (14–54)	.385
Age	<50	73 (45.6)	40 (38.1)	.225
	≥50	87 (54.4)	65 (61.69)	
T stage	1	101 (63.1)	75 (71.4)	.095
	2	57 (35.6)	30 (28.6)	
	3	2 (1.3)	0 (0.0)	
N stage	0	124 (77.5)	91 (86.7)	.091
	1	29 (18.1)	12 (11.4)	
	2	7 (4.4)	1 (1.0)	
	3	0 (0.0)	1 (1.0)	
Histologic grade	1	51 (31.9)	6 (5.7)	.001
	2	81 (50.6)	42 (40.0)	
	3	19 (11.9)	42 (40.0)	
	Unknown	9 (5.6)	15 (14.3)	
ER	Positive	135 (85.4)	47 (44.8)	.001
	Negative	23 (14.6)	58 (55.2)	
PR	Positive	122 (77.2)	41 (39.0)	.001
	Negative	36 (22.8)	64 (61.0)	
HER2	Positive	18 (11.3)	41 (39.0)	.001
	Negative	142 (88.8)	64 (61.0)	
Ki-67	<10%	66 (41.3)	36 (34.3)	.521
	10–20%	39 (24.4)	29 (27.6)	
	>30%	55 (34.4)	40 (38.1)	
NST	Yes	129 (80.6)	68 (64.8)	.004
	No	31 (19.4)	37 (35.2)	
Subtype	Luminal A	106 (66.3)	13 (12.4)	.001
	Luminal B (HER2 negative)	22 (13.8)	17 (16.2)	
	Luminal B (HER2 positive)	9 (5.6)	18 (17.1)	
	Her2 enriched	9 (5.6)	23 (21.9)	
	TNBC	14 (8.8)	34 (32.4)	
Op type	Mastectomy	43 (26.9)	19 (18.1)	.099
	BCS	117 (73.1)	86 (81.9)	

Values are expressed as mean ± standard deviation or number (%).

BCS = breast-conserving surgery, ER = estrogen receptor, HER2 = human epithelial growth factor receptor 2, NST = Neo-systemic therapy, PR = progesterone receptor, TNBC = triple negative breast cancer.

**Table 3:** Comparison of clinical and molecular characteristics between PIK3CA wild-type and mutant breast cancer.

Variables		*PIK3CA* wild-type (N = 148)	*PIK3CA* mutant (N = 117)	*P* value
Age (yr)		52.32 ± 10.26	54.17 ± 10.25	.827
Median f/u time(m)		49 (14–54)	48 (8–54)	.413
Age	<50	67 (45.3)	46 (39.3)	.33
	≥50	81 (547)	71 (60.7)	
T stage	1	92 (62.2)	84 (71.8)	.145
	2	54 (36.5)	33 (28.2)	
	3	2 (1.4)	0 (0.0)	
N stage	0	122 (82.4)	93 (79.5)	.723
	1	21 (14.2)	20 (17.1)	
	2	4 (2.7)	4 (3.4)	
	3	1 (0.7)	0 (0.0)	
Histologic grade	1	16 (10.8)	41 (35.0)	.001
	2	67 (45.3)	56 (47.9)	
	3	47 (31.8)	14 (12.0)	
	Unknown	18 (12.2)	6 (5.1)	
ER	Positive	84 (57.5)	98 (83.8)	.001
	Negative	62 (42.5)	19 (16.2)	
PR	Positive	75 (51.4)	88 (75.2)	.001
	Negative	71 (48.6)	29 (24.8)	
HER2	Positive	34 (24.3)	23 (19.7)	.356
	Negative	112 (75.7)	94 (80.3)	
Ki-67	<10%	48 (32.4)	54 (46.2)	.037
	10–20%	38 (25.7)	30 (25.6)	
	>30%	62 (41.9)	33 (28.2)	
NST	Yes	48 (32.4)	20 (17.1)	.005
	No	100 (67.6)	97 (82.9)	
Subtype	Luminal A	51 (34.5)	68 (58.1)	.001
	Luminal B (HER2 negative)	22 (14.9)	17 (14.5)	
	Luminal B (HER2 positive)	14 (9.5)	13 (11.3)	
	Her2 enriched	22 (14.9)	10 (8.5)	
	TNBC	39 (26.4)	9 (7.7)	
Op type	Mastectomy	36 (24.3)	26 (22.2)	.688
	BCS	112 (75.7)	91 (77.8)	

Values are expressed as mean ± standard deviation or number (%).

BCS = breast-conserving surgery, ER = estrogen receptor, HER2 = human epithelial growth factor receptor 2, NST = Neo-systemic therapy, PR = progesterone receptor, TNBC = triple negative breast cancer.

### 3.2. Mutational profiles

One hundred and 17 patients out of 265 had 2 or more mutations. The most common mutation was *PIK3CA,* accounting for 44.0% (n = 117), followed by *TP53* (39.5%; n = 105), *ERBB2* (13.2%; n = 35), and *GATA3* mutations (9.4%; n = 25).

**Figure 2.**
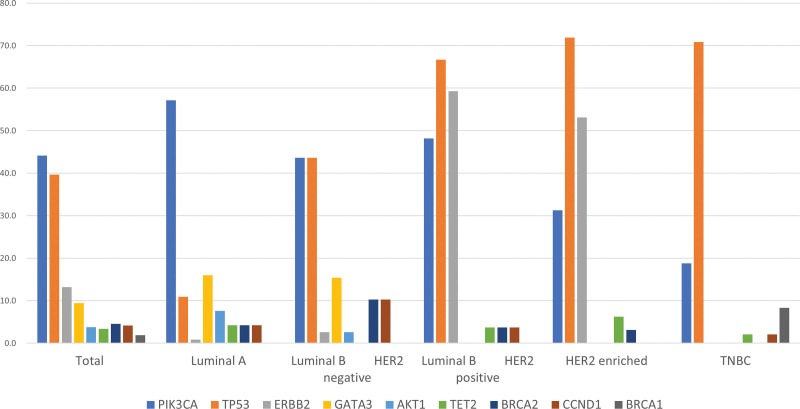
Distribution of mutations among different molecular subtypes of breast cancer. HER2 = human epithelial growth factor receptor 2, TNBC = triple negative breast cancer.

Figure 2 shows the distribution of mutations by subtype. In the luminal A type, *PIK3CA* mutations were the most common, at 57.1% (68 out of 119). In patients with luminal B and HER2-negative subtypes, *PIK3CA* and *TP53* mutations were observed in equal proportions (43.6%; 17 out of 39), while *TP53* mutations were the most common in patients with luminal B and HER2-positive subtypes (66.7%; 18 out of 27). *TP53* mutations were the most common in HER2-enriched (71.9%; 23 out of 32) and TNBC (70.8%; 34 out of 48) subtypes.

*GATA3* mutations were the 4th most common mutation and were associated with hormone receptor positivity. All 25 patients with *GATA3* mutations were hormone receptor-positive.

Patients with hormone positivity had high *PIK3CA* mutation rates, while patients with HER2 positivity and TNBC had high *TP53* mutation rates.

### 3.3. Survival analysis

During a follow-up of 48 months (range 8–54), 13 patients (4.9%) relapsed, and 3 (1.1%) died. Survival outcomes were analyzed according to mutation expression status.

**Figure 3.**
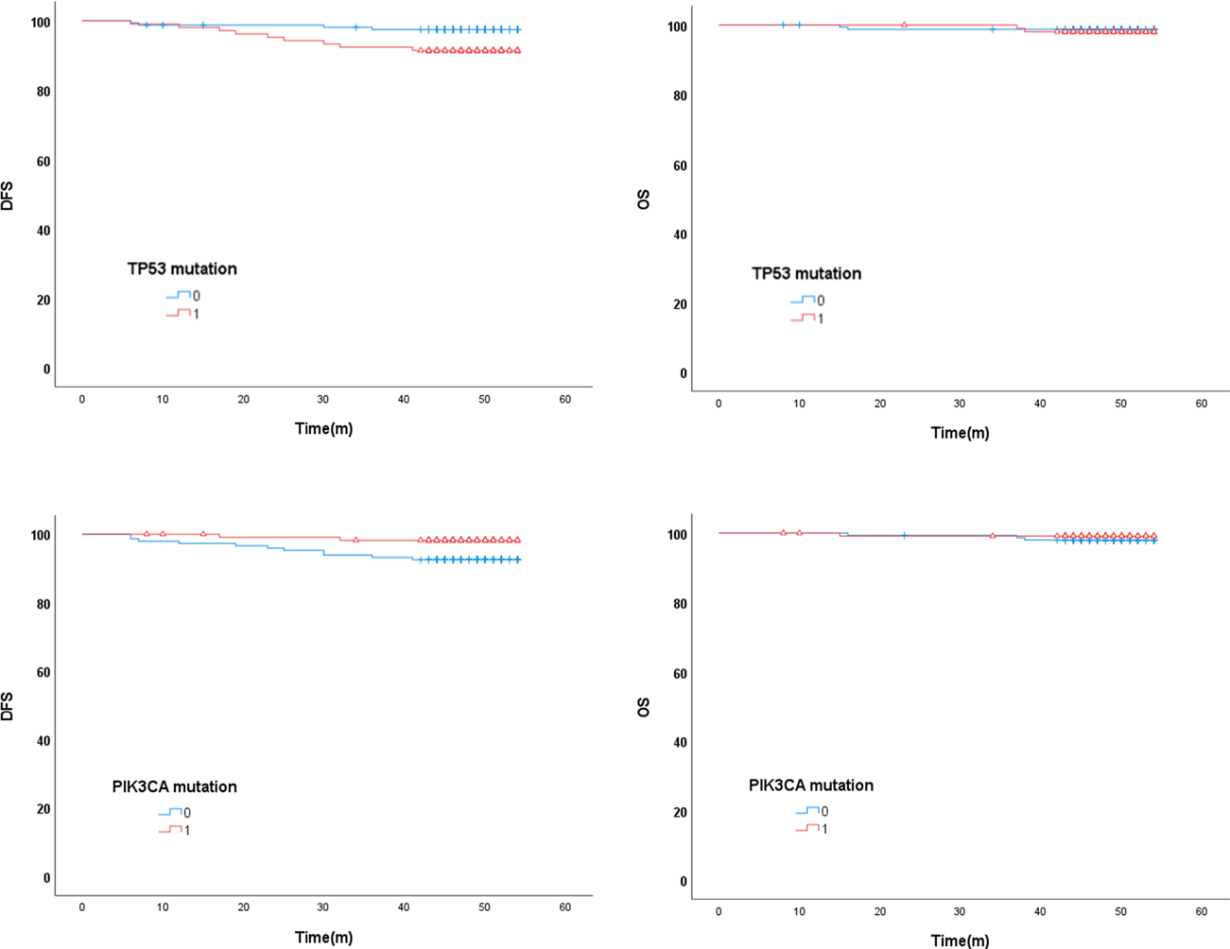
Kaplan–Meier survival analysis of the study patients according to TP53 and PIK3CA mutations. (A) The presence of TP53 mutations was associated with significantly shorter disease-free survival (DFS) (*P* = .027), but there was no significant difference in overall survival (OS) (*P* = .344). (B) On the other hand, PIK3CA mutations were associated with significantly longer DFS (*P* = .035), while no significant difference was observed in OS (*P* = .126). DFS = disease-free survival, OS = overall survival.

*TP53* mutations were significantly associated with worse short-term DFS. *PIK3CA* mutations were significantly associated with better long-term DFS. However, neither *PIK3CA* nor *TP53* mutations showed significant differences in OS (Fig. 3). For other genes, there were no significant differences in DFS or OS.

A Cox regression analysis was performed to verify the prognostic impact of *TP53* and *PIK3CA* mutations. In univariate analysis, *TP53* mutations were associated with DFS (hazard ratio, 3.468; 95% confidence interval, 1.072–11.309; *P* = .038). Additionally, patients with ER negativity showed a significant association with recurrence. However, *PIK3CA* mutations showed a marginal result in association with DFS (hazard ratio, 0.227; 95% confidence interval, 0.50–1.023; *P* = .054) (Table 4).

**Table 4:** Univariate analysis of prognostic factors affecting disease-free survival (DFS) in breast cancer patients.

Variables	Univariable analysis		
	HR	95% CI	*P* value
PIK3CA	0.227	0.50–1.023	.054
TP53	3.482	1.072–11.309	.038
T stage (T1 vs T2–3)	0.859	0.265–2.790	.8
N stage (N0 vs N1–3)	1.284	0.353–4.665	.704
ER (positive vs negative)	0.262	0.086–0.8	.019
HG (HG2,3 vs HG1)	31.093	0.141–6862.028	.212
Ki-67(≥20% vs < 20%)	2.893	0.947–8.845	.062
HER2 (positive vs negative)	1.023	0.281–3.716	.973

CI = confidence interval, DFS = disease-free survival, ER = estrogen receptor, HER2 = human epithelial growth factor receptor 2, HR = hazard ratio.

## 4. Discussion

In this study, we analyzed somatic mutations in breast cancer using NGS and investigated their correlation with clinicopathological characteristics. Our aim was to gain deeper insights into the molecular landscape of breast cancer and its implications for prognosis.

Among the somatic mutations investigated through NGS, TP53 mutations emerged as a significant poor prognostic factor, indicating a shorter DFS. These mutations were found to be associated with a high histological grade and hormone receptor negativity, both of which are well-established indicators of an unfavorable prognosis in breast cancer.

In contrast, we observed a compelling trend where PIK3CA mutations showed an association with a longer DFS. Additionally, these mutations demonstrated a favorable correlation with low histological grade, low Ki-67 level, and hormone receptor positivity, all of which are recognized as positive prognostic factors.

The tumor suppressor *p53*, which is regulated by the *TP53* gene, is critical in the prevention of cancer in normal cells.^[13]^ Many human cancers, including breast and gastric cancer, are associated with *TP53* mutations.^[14,15]^ *TP53* mutations are associated with a poor prognosis in breast cancer, especially in hormone receptor-negative and high-grade cancers.^[16]^ In our study, *TP53* mutations were associated with breast subtypes known to have a poor prognosis, such as TNBC or HER2-positive breast cancer. High histological grades and hormone receptor-negative and HER2-positive statuses were associated with a relatively short DFS. However, there was no association between tumor size and lymph node metastasis. We suggest that *TP53* mutations are associated with a poor prognosis in patients with operable breast cancer. *TP53* has also been associated with worse outcomes in hormone receptor-positive or HER2-negative and TNBC metastatic breast cancer, such as shorter survival and resistance to endocrine treatment.^[17]^ Tamoxifen is the most widely used endocrine therapy for hormone-positive breast cancer, and *TP53* mutations have been suggested to be associated with resistance to tamoxifen.^[18]^ *TP53* mutations are highly expressed in many types of cancer, including breast cancer, and are highly correlated with cancer development. Therefore, targeted therapies are highly attractive, and research on them is being actively conducted.^[19]^

In our study, *PIK3CA* mutations accounted for the largest proportion of mutations, at 44%. *PIK3CA* mutations are commonly found in hormone receptor-positive and HER2-negative breast cancer, with 57.1% of patients with hormone receptor-positive breast cancer in this study having *PIK3CA* mutations. A study that performed NGS on about 2400 people found *PIK3CA* mutations in 27% of them.^[1]^ There are several reports that have shown *PIK3CA* mutations to be associated with prognosis, with some showing better clinical outcomes than *PIK3CA* wild-type,^[20]^ while others report a poor prognosis.^[3,21]^ In our study, *PIK3CA* mutations were associated with longer DFS than *PIK3CA* wild-type. *PIK3CA* mutations were also associated with relatively low Ki-67 levels, low histological grades, and hormone receptor positivity, and relatively few TNBC-type breast cancers were reported in this group. Alpelisib, which targets *PIK3CA* mutations, has been developed and used in combination with fulvestrant for the treatment of hormone-positive and HER2-negative metastatic breast cancer, improving progression-free survival and overall response better than fulvestrant alone.^[3,22]^

*ERBB2* amplification and HER2 overexpression have been the most extensively studied in breast cancer. *ERBB2* amplification is the most common mechanism leading to increased HER2 protein overexpression.^[23]^ HER2, encoded by *ERBB2*, is an important member of the receptor tyrosine kinase family, which is activated by homo- or heterodimer formation with other *ERBB* family receptors.^[24]^ *HER2* amplification is a prognostic biomarker for worse survival in the absence of anti-HER2 therapy.^[25]^ Traditionally, *HER2* overexpression or amplification is assessed by IHC or FISH rather than NGS,^[26]^ and there are differences between traditional methods and NGS results for the detection of HER2 overexpression and amplification.^[27]^ In this study, *ERBB2* mutations were also observed in most patients with HER2-positive breast cancer (33/35, 94.3%), but not in all patients with HER2-positive breast cancer (33/59, 55.9%). Further research is required to confirm this hypothesis.

The *GATA3* protein plays an important role in cell development and differentiation in various types of cells, including breast tissue.^[28]^ Several studies have reported that *GATA3* mutations are associated with improved survival rates in patients with breast cancer. However, in our study, *GATA3* mutations were the 4th most common mutation after *PIK3CA, TP53,* and *ERBB2* mutations. *GATA3* mutations were not associated with DFS.^[29]^ It has been reported that *GATA3* mutations are strongly associated with the ER but lack value as an independent prognostic factor and are not useful for predicting endocrine therapy.^[30]^ The value of *GATA3* mutations as prognostic factors remains unclear and requires further study.

In this study, NGS was performed for all breast cancers that had surgery at a single institution over the course of a year. Molecular subtype analysis of naturally occurring breast cancer aids in the classification of breast cancer in each region.

Our study has several limitations. First, as it is a retrospective study, there are limitations to the analysis because it relies on medical records. Second, our panel contained only 143 genes, and some gene mutations were not detected. Third, the observation period was short, so it was not possible to determine whether recurrence was delayed after 5 years. Fourth, survival analysis could not be performed according to subtype. Fifth, we found an association between *PIK3CA* mutations and a longer DFS, but the univariate analysis of DFS showed a marginal result with a *P* value of .054. This is thought to be because the number of cases and events was small and the observation period was short because the study was conducted with only a patient group recruited for 1 year at a single institution.

Notwithstanding these limitations, our findings indicate that TP53 mutations, among somatic mutations, serve as independent prognostic factors for breast cancer. Additionally, we observed an association between PIK3CA mutations and a favorable trend in disease-free survival. This study provides information on the molecular profiles of breast cancer and helps to predict the prognosis. This will also provide information for the development of therapeutics that target somatic mutations.

## 5. Conclusion

We found that TP53 mutations are associated with a poor prognosis, while PIK3CA mutations show a tendency towards a favorable prognosis. These results provide valuable insights for breast cancer treatment and suggest that NGS analysis of somatic mutations can be a useful tool for guiding therapeutic strategies and prognostic evaluations.

These findings contribute to a better understanding of the molecular profiles of breast cancer and hold potential implications for advancing precision medicine. We hope that this research will encourage further studies in breast cancer treatment and prognosis improvement, making significant contributions to the fields of medical and pharmaceutical development.

## Acknowledgments

We would like to thank Editage (www.editage.co.kr) for English language editing.

## Author contributions

**Conceptualization:** Jin Hyuk Chio, Sung Ui Jung.

**Data curation:** Jin Hyuk Chio, Jesang Yu, Junyong Jekal, Sung Ui Jung.

**Formal analysis:** Jin Hyuk Chio, Jesang Yu, Sung Ui Jung.

**Funding acquisition:** Sung Ui Jung.

**Investigation:** Jin Hyuk Chio, Sung Ui Jung.

**Methodology:** Jesang Yu, Minjung Jung, Sung Ui Jung.

**Project administration:** Junyong Jekal, Sung Ui Jung.

**Resources:** Jin Hyuk Chio, Sung Ui Jung.

**Software:** Sung Ui Jung.

**Supervision:** Ku Sang Kim, Sung Ui Jung.

**Validation:** Ku Sang Kim, Sung Ui Jung.

**Visualization:** Ku Sang Kim, Sung Ui Jung.

**Writing – original draft:** Jin Hyuk Chio, Jesang Yu, Sung Ui Jung.

**Writing – review & editing:** Sung Ui Jung.
